# Loss of Basal Forebrain Cholinergic Neurons Following Adolescent Binge Ethanol Exposure: Recovery With the Cholinesterase Inhibitor Galantamine

**DOI:** 10.3389/fnbeh.2021.652494

**Published:** 2021-02-26

**Authors:** Fulton T. Crews, Rachael Fisher, Chloe Deason, Ryan P. Vetreno

**Affiliations:** ^1^Bowles Center for Alcohol Studies, School of Medicine, University of North Carolina at Chapel Hill, Chapel Hill, NC, United States; ^2^Department of Psychiatry, School of Medicine, University of North Carolina at Chapel Hill, Chapel Hill, NC, United States

**Keywords:** adolescence, alcohol, binge drinking, epigenetic, neuroimmune, choline acetyltransferase

## Abstract

Binge drinking and alcohol abuse are common during adolescence and cause both cognitive deficits and lasting cholinergic pathology in the adult basal forebrain. Acetylcholine is anti-inflammatory and studies using the preclinical adolescent intermittent ethanol (AIE; 5.0 g/kg, i.g., 2 day on/2 day off from postnatal day [P]25 to P54) model of human adolescent binge drinking report decreased basal forebrain cholinergic neurons (BFCNs) and induction of proinflammatory genes that persist long into adulthood. Recent studies link AIE-induced neuroimmune activation to cholinergic pathology, but the underlying mechanisms contributing to the persistent loss of BFCNs are unknown. We report that treatment with the cholinesterase inhibitor galantamine (4.0 mg/kg, i.p.) administered during AIE (i.e., P25–P54) or following the conclusion of AIE (i.e., P57–P72) recovered the persistent loss of cholinergic neuron phenotype markers (i.e., ChAT, TrkA, and p75^NTR^) and somal shrinkage of residual ChAT + neurons known to persist in AIE-exposed adults. Galantamine treatment also recovered the AIE-increased expression of the proinflammatory receptors TLR4 and RAGE, the endogenous TLR4/RAGE agonist HMGB1, and the transcription activation marker pNF-κB p65. Interestingly, we find BFCNs express TLR4 and RAGE, and that AIE treatment increased pNF-κB p65 expression in adult ChAT + IR neurons, consistent with intracellular HMGB1-TLR4/RAGE signaling within BFCNs. AIE increased epigenetic transcription silencing markers (i.e., H3K9me2 and H3K9me3) in the adult basal forebrain and H3K9me2 occupancy at cholinergic phenotype gene promoters (i.e., *ChAT* and *TrkA*). The finding of no AIE-induced changes in total basal forebrain NeuN + neurons with galantamine reversal of AIE-induced ChAT + neuron loss, TLR4/RAGE-pNF-κB p65 signals, and epigenetic transcription silencing markers suggests that AIE does not cause cell death, but rather the loss of the cholinergic phenotype. Together, these data suggest that AIE induces HMGB1-TLR4/RAGE-pNF-κB p65 signals, causing the loss of cholinergic phenotype (i.e., ChAT, TrkA, and p75^NTR^) through epigenetic histone transcription silencing that result in the loss of the BFCN phenotype that can be prevented and restored by galantamine.

## Introduction

Adolescence is a conserved neurodevelopmental period characterized by increased social interactions and risky behavior that coincide with refinement of brain neurotransmitter systems (Shoval et al., 2014), including the cholinergic system of the basal forebrain (Semba, 2004; Coleman et al., 2011), that parallel the transition from adolescent to adult characteristic behaviors (Spear, 2009). Basal forebrain cholinergic neurons (BFCNs) are critically involved in cognitive function and modulation of cortical networks (Ballinger et al., 2016; Blake and Boccia, 2017; Peeters et al., 2020) due to their extensive projections to the hippocampus and multiple cortical regions (Mesulam et al., 1983; Baxter and Chiba, 1999). Adolescent binge drinking is associated with lasting consequences that persist into adulthood, including increased risk for alcohol use disorder (AUD) (Grant and Dawson, 1997) and neurodegenerative diseases, such as Alzheimer’s disease (AD) (Viner and Taylor, 2007; Kamal et al., 2020). Indeed, degeneration of BFCNs and ChAT + somal shrinkage are features of several neurodegenerative disorders, including AD and AUD (Lehericy et al., 1993; Vetreno et al., 2014), and may contribute to the cognitive deficits associated with these disorders (Vogels et al., 1990; De Rosa et al., 2004; Mufson et al., 2008), all of which are also associated with increased expression of neuroimmune genes. Employing the preclinical adolescent intermittent ethanol (AIE) model of human adolescent binge drinking, we find that AIE, but not an identical adult intermittent ethanol treatment, causes long-lasting partial reductions of BFCN markers (i.e., choline acetyltransferase [ChAT] and vesicular acetylcholine transporter [VAChT] as well as the nerve growth factor [NGF] receptors tropomyosin receptor kinase A [TrkA] and p75 neurotrophin receptor [p75^NTR^]) that are accompanied by somal shrinkage of the remaining ChAT + neurons. Studies in adult AIE-treated rats as well as in human AD and AUD find reductions of BFCNs and increased expression of proinflammatory neuroimmune signals, including Toll-like receptor 4 (TLR4), receptor for advanced glycation end-products (RAGE), and the endogenous TLR4/RAGE cytokine-like agonist high-mobility group box 1 (HMGB1) (Crews et al., 2013; Vetreno et al., 2013; Paudel et al., 2020). HMGB1 binds to and activates TLR4, RAGE, and other receptors, leading to nuclear translocation of NF-κB, thereby contributing to complex proinflammatory signaling (Mayfield et al., 2013; Crews et al., 2017b). The AIE-induced loss of BFCNs is accompanied by persistent increases of HMGB1, TLR4, RAGE, phosphorylated (activated) NF-κB p65 (pNF-κB p65), and downstream proinflammatory neuroimmune genes throughout the adult brain, including the prefrontal cortex and hippocampus (Vetreno and Crews, 2012; Vetreno et al., 2013, 2018; Vetreno and Crews, 2018). In rodent studies, administration of the TLR4 ligand lipopolysaccharide (LPS) induces lasting upregulation of proinflammatory NF-κB target genes and reductions of BFCNs (Qin et al., 2007, 2008; Vetreno and Crews, 2018). The anti-inflammatory drug indomethacin and voluntary wheel running exercise exposure during AIE treatment block the AIE increase of proinflammatory genes and prevent BFCN ChAT immunoreactive (+ IR) loss, consistent with neuroimmune signals contributing to reductions of BFCNs (Vetreno and Crews, 2018). However, it is possible that the loss of anti-inflammatory acetylcholine also contributes to increases in neuroimmune signaling. In the hippocampus, AIE induces HMGB1, RAGE, TLRs, and other neuroimmune genes in association with reductions of adult neurogenesis (Crews et al., 2017b; Vetreno et al., 2018). Recent studies find that the AIE-induced loss of hippocampal neurogenesis and neuroimmune gene induction are blocked by exercise, indomethacin, and donepezil, which is a cholinesterase inhibitor used to treat AD (Vetreno et al., 2018; Swartzwelder et al., 2019). In this study, we extend these findings to BFCNs and find that AIE combined with the cholinesterase inhibitor galantamine prevents the AIE induction of adult neuroimmune signaling molecules and loss of BFCNs.

Emerging studies suggest neuroimmune induction and ethanol exposure can elicit long-lasting changes in chromatin programming through chromatin remodeling (Montesinos et al., 2016; Chastain and Sarkar, 2017; Pandey et al., 2017; Wolstenholme et al., 2017; Nestler and Luscher, 2019; Vetreno et al., 2019). Epigenetic modifications involve histone acetylation and methylation, which can enhance or repress gene transcription without changing the underlying DNA sequence (Kouzarides, 2007; Berger et al., 2009; Fernandes et al., 2017). Acetylation and methylation of histone 3 lysine 9 (H3K9) can activate and repress gene transcription, respectively (Wang et al., 2008). Recent studies have found that AIE exposure increases adult anxiety as well as alcohol drinking and preference through epigenetic programming within the amygdala that is reversed with inhibitors and other molecular tools (Kyzar et al., 2016; Pandey et al., 2017). Similarly, AIE-induced loss of BFCN markers is accompanied by increased histone 3 acetyl 9 dimethylation (H3K9me2) occupancy at cholinergic gene promoters that is reversed by post-AIE exercise exposure (Vetreno et al., 2019). In hippocampus, Swartzwelder et al. (2019) found AIE-induced reductions of hippocampal neurogenesis are accompanied by increased expression of RAGE and pNF-kBp65 as well as the transcriptional-silencing marker H3K9me2 that is reversed by treatment with the cholinesterase inhibitor donepezil in adulthood. Although loss of cholinergic neurons was initially thought to involve ChAT + neuron loss and cognitive deficits, our exercise reversal studies support a reversal of epigenetic programming. Interestingly, early studies of fimbria-fornix lesion-induced loss of BFCNs and cholinergic neuron shrinkage found NGF infusions reverse pathology (Hagg et al., 1988, 1989). We therefore tested the hypothesis that treatment with galantamine, which is a cholinesterase inhibitor with FDA approval for the treatment of AD (Lilienfeld, 2002; Hampel et al., 2018; Haake et al., 2020), would recover the AIE-induced cholinergic pathology in the adult basal forebrain. We find that galantamine treatment following AIE recovers the loss of cholinergic neuron markers (i.e., restores ChAT, TrkA, and p75^NTR^ expression) and restores the large BFCN somal size. These findings provide additional support for epigenetic programming having a long-lasting impact on brain function and behavior, with the exciting possibility that neurobiological changes may be reversible.

## Materials and Methods

### Animals

Male Wistar rats bred and reared at the University of North Carolina at Chapel Hill were used in this study. We previously reported that AIE treatment, which models human adolescent binge drinking, causes a persistent reduction of ChAT (BFCNs in both male and female rats (Vetreno et al., 2014, 2019; Vetreno and Crews, 2018). On the day following birth (postnatal day [P]1), litters remained with their dams in standard clear plastic tubs with shavings until group housing with same-sex littermates at the time of weaning on P21. Subjects were housed in a temperature −20°C) and humidity-controlled vivarium on a 12/12 h light/dark cycle (light onset at 7:00 a.m.), and provided *ad libitum* access to food and water. This study was performed in an AAALAC-accredited facility and conducted in strict accordance with NIH regulations for the care and use of animals in research. Experimental procedures reported in this study were approved by the Institutional Animal Care and Use Committee of the University of North Carolina at Chapel Hill (Protocol #: 20-189).

### Adolescent Intermittent Ethanol (AIE) Paradigm

On P21, Wistar rats (*N* = 108) were randomly assigned to either: (i) AIE (*n* = 54) or (ii) water control (CON; *n* = 54) conditions. To minimize the impact of litter variables, no more than one subject from a given litter was assigned to a single experimental condition. From P25 to P54, AIE subjects received a single daily intragastric (i.g.) administration of ethanol (EtOH; 5.0 g/kg, 25% EtOH, w/v) in the morning on a 2 day on/2 day off schedule, and CON subjects received comparable volumes of water on an identical schedule as previously described (Vetreno et al., 2019). Body weight increases dramatically during rat adolescence (i.e., approximately fourfold [∼80 to 400 g]), so we treat with g/kg to match body weight. Tail blood was collected from AIE- and CON-treated subjects 1 h after treatment on P38 and P54 to assess blood ethanol concentrations (BECs) using a GM7 Analyzer (Analox; London, United Kingdom). Body weights were assessed through AIE to the conclusion of experimentation.

### Galantamine Treatment

**FIGURE 1:**
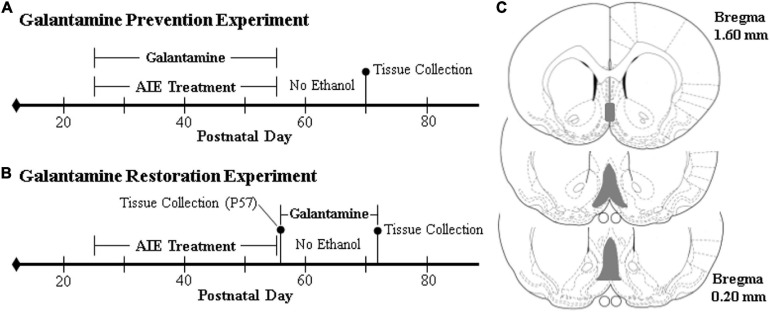
Schematic of the galantamine prevention and restoration experimental design. On postnatal day (P)21, male Wistar rats were randomly assigned to either (i) water control (CON) or (ii) adolescent intermittent ethanol (AIE) treatment conditions. From P25 to P54, AIE subjects received a single daily intragastric (i.g.) gavage administration of ethanol (EtOH; 5.0 g/kg, 25% EtOH, w/v) in the AM on a 2 day on/2 day off schedule, and CONs received comparable volumes of water on an identical schedule. **(A)** In the galantamine prevention experiment, adolescent CON and AIE subjects received a single dose of galantamine HCl (4.0 mg/kg, i.p. in sterile PBS) or vehicle 30 min prior to each EtOH administration from P25 to P54. At the conclusion of treatment on P54, subjects were left undisturbed until sacrifice on P70. **(B)** In the galantamine restoration experiment, adult CON- and AIE-treated subjects received a single daily dose of galantamine HCl (4.0 mg/kg, i.p. in sterile PBS) or vehicle beginning 72 h following the conclusion of AIE treatment from P57 to P72. At the conclusion of treatment, subjects were sacrificed 24 h later on P73. **(C)** Depicted are coronal sections of the medial septum and vertical limb of the diagonal band of the basal forebrain between Bregma 1.60 and 0.20 mm according to the atlas of Paxinos and Watson (1998) used for immunohistochemical analysis. Gray shaded regions indicate regions of interest.

To determine if the cholinesterase inhibitor galantamine prevents or restores AIE-induced basal forebrain neuropathology, subjects received treatment with galantamine HCl (4.0 mg/kg, i.p. in sterile phosphate-buffered saline [PBS]; Sigma-Aldrich, St. Louis, MO, Cat. #1287755) or vehicle (sterile PBS) during AIE treatment (preventative) or post-AIE treatment (restorative). In the galantamine prevention experiment (*N* = 32; *n* = 8 subjects per group), CON- and AIE-treated subjects received a single dose of galantamine or vehicle 30 min prior to each EtOH administration from P25 to P54 (see Figure 1A). Treatment with galantamine during AIE did not affect BECs (± SEM) at either P38 (AIE/Vehicle: 172 mg/dL [±27], AIE/Galantamine: 158 mg/dL [±25]; Student’s *t*-test, *p* > 0.70) or P54 (AIE/Vehicle: 124 mg/dL [±23], AIE/Galantamine: 150 mg/dL [±13]; Student’s *t*-test, *p* > 0.30). While subjects in the galantamine prevention experiment evidenced dramatic body weight increases across age (main effect of Age: *p* < 0.01, repeated measures ANOVA), we did not observe an effect of AIE (main effect of Treatment: *p* > 0.30, repeated measures ANOVA) or galantamine (main effect of Drug: *p* > 0.20) on body weight.

In the galantamine restoration experiment (*N* = 76; *n* = 38 subjects per treatment), CON- and AIE-treated subjects (*N* = 60; *n* = 15 subjects per group) received a single dose of galantamine or vehicle daily from P57 to P72 (see Figure 1B). The remainder of subjects (*N* = 16; n = 8/CON, *n* = 8/AIE) were sacrificed on P57 to verify ChAT + loss following AIE. Prior to galantamine treatment in the restoration experiment, there were no differences in BECs (± SEM) at either P38 (AIE/Vehicle: 142 mg/dL [±22], AIE/Galantamine: 171 mg/dL [±18]; Student’s *t*-test, *p* > 0.30) or P54 (AIE/Vehicle: 188 mg/dL [±25], AIE/Galantamine: 195 mg/dL [±40]; Student’s *t*-test, *p* > 0.80). While subjects in the galantamine restoration experiment evidenced dramatic body weight increases across age (main effect of Age: *p* < 0.01, repeated measures ANOVA), we did not observe an effect of AIE (main effect of Treatment: *p* > 0.07, repeated measures ANOVA) or galantamine (main effect of Drug: *p* > 0.82) on body weight. Across studies, CONs did not evidence detectable BECs, consistent with water treatment.

### Perfusion and Brain Tissue Preparation

Subjects were sacrificed on P70 (prevention experiment) or P73 (restoration experiment), and brain tissue collected for assessment. At the conclusion of each experiment, subjects were anesthetized with a lethal dose of sodium pentobarbital (100 mg/kg, i.p.). For immunohistochemical studies, rats (*n* = 8 subjects per group) were transcardially perfused with 0.1 M PBS followed by 4.0% paraformaldehyde in PBS. Brains were excised and post-fixed in 4.0% paraformaldehyde for 24 h at 4°C followed by 4 d fixation in 30% sucrose solution. Coronal sections were cut (40 μm) on a sliding microtome (MICROM HM450; ThermoScientific, Austin, TX), and sections sequentially collected into well plates and stored at −20°C in a cryoprotectant solution (30% glycol/30% ethylene glycol in PBS). For DNA extraction, rats in the galantamine restoration experiment (*n* = 7 subjects per group) were transcardially perfused with 0.1 M PBS, and basal forebrain tissue dissected, rapidly frozen in liquid nitrogen, and stored at −80°C.

### Immunohistochemistry (IHC)

Free-floating basal forebrain tissue samples (every 6th section; approximate Bregma: 1.60–0.20 mm based on the atlas of Paxinos and Watson, 1998; see Figure 1C) were washed in 0.1 M PBS, incubated in 0.6% H_2_O_2_ to inhibit endogenous peroxidases, and blocked with normal serum (MP Biomedicals, Solon, OH). Sections were then incubated in a primary antibody solution for 24 h at 4°C. Primary antibodies, dilutions, and validation information are included in Table 1. Sections were washed with PBS, incubated for 1 h in a biotinylated secondary antibody (Vector Laboratories, Burlingame, CA), and incubated for 1 h in avidin-biotin complex solution (Vectastain ABC Kit; Vector Laboratories). The chromogen, nickel-enhanced diaminobenzidine (Sigma-Aldrich), was used to visualize immunoreactivity. Tissue was mounted onto slides, dehydrated, and cover slipped. Negative control for non-specific binding was conducted on separate sections employing the abovementioned procedures omitting the primary antibody.

**TABLE 1:** List of primary antibodies used for immunohistochemistry.

Antibody	Isotype	Dilution	Source/purification	Company, catalog number	Validation
ChAT	Goat IgG	1:200	Polyclonal	Millipore, AB144P	WB (Millipore)
TrkA	Rabbit IgG	1:200	Polyclonal	Millipore, 06-574	WB (Millipore)
p75^NTR^	Mouse IgG	1:200	Monoclonal	Millipore, MAB365	Binding assay^a^
HMGB1	Rabbit IgG	1:1,000	Polyclonal	Abcam, ab18256	KO Validated (Abcam)
TLR4	Mouse IgG	1:100	Monoclonal	Abcam, ab22048	KO Validated (Abcam)
RAGE	Rabbit IgG	1:1,000	Polyclonal	Abcam, ab3611	WB (Abcam)
NF-κB p65 (phospho S536)	Rabbit IgG	1:2,000	Polyclonal	Abcam, ab86299	WB (Abcam)
H3K9ac	Rabbit IgG	1:500	Polyclonal	Abcam, ab10812	WB/Peptide validated (Abcam)
H3K9me2	Mouse IgG	1:1,000 (IHC) 5 μL/sample (ChIP)	Monoclonal	Abcam, ab1220	WB (Abcam)
H3K9me3	Rabbit IgG	1:300	Polyclonal	Abcam, ab8898	WB/Peptide validated (Abcam)
NeuN	Mouse IgG	1:500	Monoclonal	Millipore, MAB377	IHC (Millipore)

*Validation information includes references as well as methods used for validation and source.*

*^a^Chandler et al. (1984).*

### Microscopic Quantification and Image Analysis

Across experiments, BioQuant Nova Advanced Image Analysis software (R&M Biometric, Nashville, TN) was used for image capture and quantification of IHC. Representative images were captured using an Olympus BX50 microscope and Sony DXC-390 video camera linked to a computer. For each measure, the microscope, camera, and software were background corrected and normalized to preset light levels to ensure fidelity of data acquisition. Microscopic quantification, which was performed by experimenters blind to treatment conditions, was conducted in the medial septum and vertical limb of the diagonal band, as we previously discovered that AIE causes a persistent loss of ChAT + IR cells throughout the cholinergic nuclei of the brain (Vetreno et al., 2014). A modified unbiased stereological quantification method was used to quantify immunoreactivity in the basal forebrain. While there is variability in immunoreactive cell density across basal forebrain coronal position, IHC staining for all groups within studies were processed at the same time with identical coronal sections to reduce variability. However, although baseline values of basal forebrain IHC staining vary across studies and cohorts, this variation does not affect group comparisons. We previously reported that comparison of traditional unbiased stereological methodology with our modified unbiased stereological approach yielded nearly identical values relative to CONs (Crews et al., 2004). The outlined regions of interest were determined and data expressed as cells/mm^2^. ChAT (+IR somal size was assessed using BioQuant Nova Advanced Image Analysis software (R&M Biometric).

### Fluorescent IHC and Microscopy

To assess ChAT colocalization with pNF-κB p65, TLR4, and RAGE, free-floating basal forebrain sections were processed similar to previously reported methods (Vetreno et al., 2019). Briefly, sections were washed in 0.1 M Tris-buffered saline (TBS), antigen retrieval performed by incubation in Citra solution (BioGenex, Fremont, CA) for 1 h at 70°C, and blocked with normal horse serum (MP Biomedicals). Sections were incubated for 48 h at 4°C in a primary antibody cocktail of goat anti-ChAT (Millipore) in combination with either mouse anti-TLR4 (Abcam), rabbit anti-RAGE (Abcam), or rabbit anti-pNF-κB p65 (Abcam) (see Table 1). Sections were washed in TBS and incubated for 2 h at room temperature in a secondary antibody cocktail (Alexa Fluor 594, Alexa Fluor 488; Invitrogen, Carlsbad, CA). Tissue was mounted onto slides and cover slipped using Prolong Gold Anti-Fade mounting media (Life Technologies, Grand Island, NY). Immunofluorescent images were obtained using a DS-RiZ scope (Nikon Inc., Melville, NY) and colocalization quantified using NIS Elements AR46 (Nikon Inc.).

### Chromatin Immunoprecipitation (ChIP)

The procedure used is similar to methods reported previously (Kyzar et al., 2017; Vetreno et al., 2019). Briefly, basal forebrain tissue samples from vehicle- and galantamine-treated CON- and AIE-treated subjects (*n* = 7 subjects per group) in the galantamine restoration study were homogenized, fixed in 1.0% methanol-free formaldehyde, quenched with 1.0 M glycine, lysed with lysis buffer (1.0% [v/v] SDS, 10 mM EDTA, 50 mM Tris-HCl [pH 8.0]), and chromatin sheared to fragments of < 1,000 bp on a Covaris ME220. Input DNA fractions were removed from the sheared chromatin to be processed separately and the remaining sheared chromatin was incubated overnight at 4°C with an antibody against H3K9me2 (5 μl/sample; Abcam) (see Table 1). Protein A Dynabeads (ThermoFisher Scientific, Austin, TX) were added and rotated at 4°C for 1 h followed by six washes in ChIP wash buffer. Both immunoprecipitated DNA and input DNA were eluted in 10% (w/v) Chelex by boiling at 95°C for 10 min followed by centrifugation. The resulting DNA was quantified using qPCR with SSOAdvanced Universal SYBR Green Supermix (Bio-Rad, Berkeley, CA) using primers targeted against the *ChAT* and *TrkA* promoters (see Table 2). The ΔΔCt method was used to determine fold change relative to CON and was normalized to the Input DNA fraction.

**TABLE 2:** List of primer sequences for ChIP analysis.

Primer	Forward	Reverse
*ChAT* Promoter	ACT TGA TTG CTG CCT CTC TC	GGG ATG GTG GAA GAT ACA GAA G
*ChAT* Promoter CpG Island	TGC ATC TGG AGC TCA AAT CGT	GGG GAT AGT GGT GAC GTT GT
*TrkA* Promoter	CCT CAC CGT GCA CTT TAC CT	AGG GTC TGG AGA GCG TAC AT
*TrkA* Promoter CpG Island	TCA AGC AAG GCT CCG AAC AG	CAC AGG GTG GCG CTA GAA G

### Statistical Analysis

Statistical analysis was performed using GraphPad Software (Prism 9; San Diego, CA). Two-tailed Student’s *t*-tests were used to assess BECs (P38 and P54) and ChAT + IR at P57. Repeated measure ANOVAs were used to assessed body weight. The immunohistochemical and ChIP data were analyzed using 2 × 2 ANOVAs. *Post hoc* analyses were performed using Tukey’s HSD where appropriate. Significant interactions are discussed in relation to outcomes of Tukey’s HSD *post hoc* tests. All values are reported as mean ± SEM, and significance was defined as *p* ≤ 0.05.

## Results

### Adolescent Galantamine Treatment Combined With AIE Prevents the Loss of Cholinergic Neuron Markers and Shrinkage of Residual ChAT+ Neurons in the Adult Basal Forebrain

**FIGURE 2:**
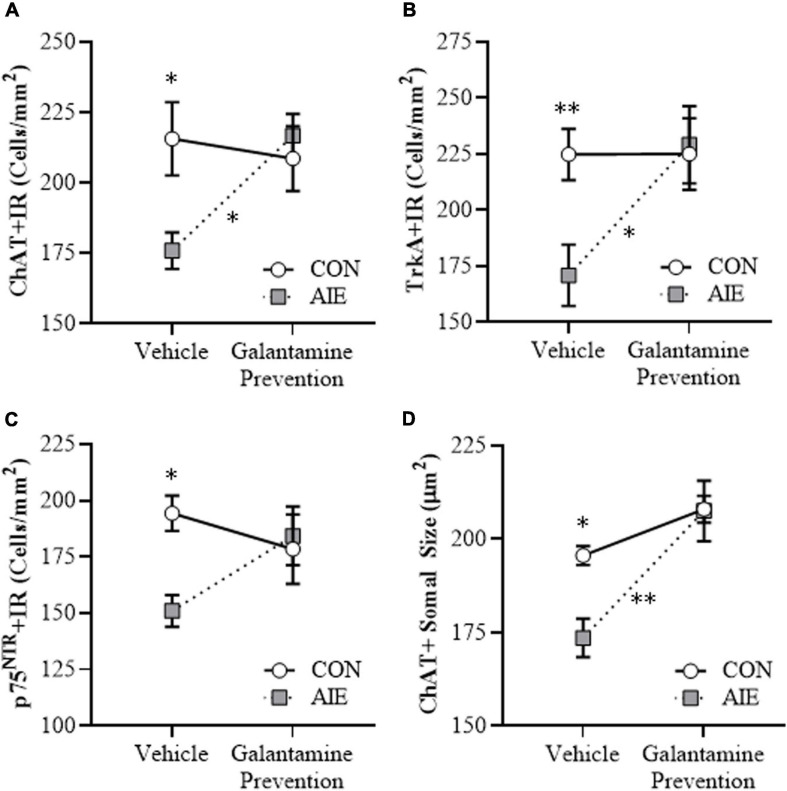
Adolescent galantamine treatment prevented the adolescent intermittent ethanol (AIE)-induced loss and somal shrinkage of cholinergic neurons in the adult basal forebrain. **(A)** Modified unbiased stereological assessment revealed an 18% (±3%) reduction of choline acetyltransferase immunoreactive (ChAT + IR) neurons in the adult (P70) basal forebrain of AIE-treated subjects, relative to CONs. Adolescent galantamine treatment from P25 to P54 did not affect ChAT + IR in CONs, but prevented the AIE-induced loss of ChAT + IR neurons in adulthood, relative to vehicle-treated AIE subjects. **(B)** Modified unbiased stereological assessment revealed a 24% (±6%) reduction of tropomyosin receptor kinase A immunoreactive (TrkA + IR) neurons in the adult (P70) basal forebrain of AIE-treated subjects, relative to CONs. Adolescent galantamine treatment from P25 to P54 did not affect TrkA + IR in CONs, but prevented the AIE-induced loss of TrkA + IR neurons in adulthood, relative to vehicle-treated AIE subjects. **(C)** Modified unbiased stereological assessment revealed a 22% (±4%) reduction of p75 neurotrophin receptor immunoreactive (p75^NTR^ + IR) neurons in the adult (P70) basal forebrain of AIE-treated subjects, relative to CONs. Adolescent galantamine treatment from P25 to P54 did not affect p75^NTR^ + IR in CONs and blunted the AIE-induced loss of p75^NTR^ + IR neurons in adulthood, relative to vehicle-treated AIE subjects. **(D)** Analysis of ChAT + IR neuron somal size revealed an 11% (±3%) reduction in somal size of the residual ChAT + neurons in the adult basal forebrain of AIE-treated subjects, relative to CONs. Galantamine treatment alone did not affect ChAT + somal size, but prevented the AIE-induced somal shrinkage of ChAT + cholinergic neurons in the adult basal forebrain, relative to vehicle-treated AIE subjects. Data are presented as mean ± SEM (n = 8/group). ^∗^*p* < 0.05, ^∗∗^*p* < 0.01.

BFCNs are characterized by expression of ChAT and the NGF receptors TrkA and p75^NTR^ (Sobreviela et al., 1994; Vetreno and Crews, 2018; Vetreno et al., 2019). Our laboratory and others previously reported that populations and size of BFCNs do not differ between male and female rats, and that AIE treatment causes a comparable loss of BFCNs across sexes in adulthood (Gibbs, 1998; Vetreno and Crews, 2018). In the present study, we sought to determine if treatment during AIE (i.e., P25–P54) with the cholinesterase inhibitor galantamine, which is approved for the treatment of AD (Lilienfeld, 2002; Haake et al., 2020), would prevent the loss of BFCN markers in the adult (i.e., P70) basal forebrain. We report an 18% (±3%) reduction of ChAT + IR (Tukey’s HSD: *p* < 0.05), a 24% (±6%) reduction of TrkA + IR [one-way ANOVA: *F*_(__1_, _14__)_ = 9.2, *p* < 0.01], and a 22% (±4%) reduction of p75^NTR^ + IR (Tukey’s HSD: *p* < 0.05) cells in the basal forebrain of adult AIE-treated subjects, relative to CON subjects (see Figures 2A–C). Adolescent galantamine treatment alone did not affect expression of BFCN markers in CONs but prevented the AIE-induced loss of ChAT + IR (Tukey’s HSD: *p* < 0.05) and TrkA + IR cells [one-way ANOVA: *F*_(__1_, _14__)_ = 7.0, *p* < 0.05], and blunted the loss of p75^NTR^ + IR cells in the adult basal forebrain, relative to vehicle-treated AIE subjects. Assessment of ChAT + BFCNs revealed a significant AIE-induced 11% (±3%) reduction in somal size of the residual ChAT + IR neurons in the adult basal forebrain (Tukey’s HSD: *p* < 0.05), relative to CON subjects (see Figure 2D). Similar to the BFCN markers, adolescent galantamine treatment alone did not affect ChAT + somal size in CONs, but prevented the AIE-induced somal shrinkage of ChAT + IR BFCNs in the adult basal forebrain (Tukey’s HSD: *p* < 0.01), relative to vehicle-treated AIE subjects. Together, these data indicate that treatment with galantamine during AIE provided long-lasting prevention of the BFCN marker loss and ChAT + somal shrinkage in the adult basal forebrain.

### Adolescent Galantamine Treatment During AIE Prevents the AIE-Induced Increases of Proinflammatory Neuroimmune Signals and NF-κB activation Within Adult BFCNs

**FIGURE 3:**
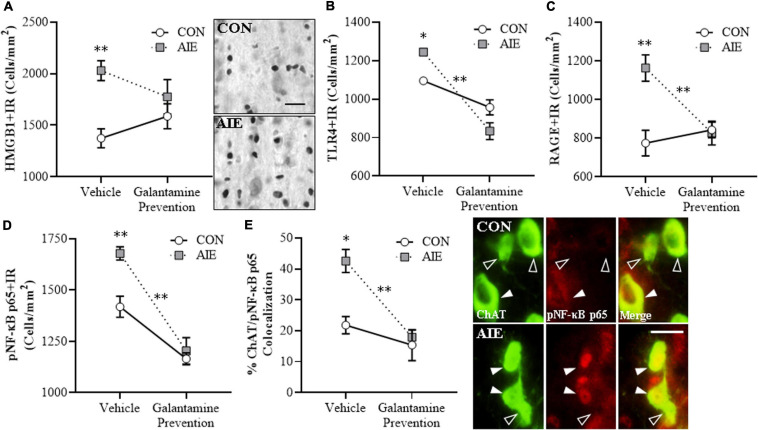
Adolescent galantamine treatment prevented the adolescent intermittent ethanol (AIE)-induced increase of proinflammatory neuroimmune signaling molecules in the adult basal forebrain. **(A)** Modified unbiased stereological assessment revealed a 48% (±7%) increase of high-mobility group box 1 immunoreactive (HMGB1 + IR) cells in the adult (P70) basal forebrain of AIE-treated subjects, relative to CONs. Adolescent galantamine treatment from P25 to P54 did not affect HMGB1 + IR in CONs, but blunted the AIE-induced increase of HMGB1 + IR cells in adulthood, relative to vehicle-treated AIE subjects. Representative photomicrographs of HMGB1 + IR cells in the adult basal forebrain of CON- and AIE-treated subjects. Scale bar = 50 μm. **(B)** Modified unbiased stereological assessment revealed a 14% (±2%) increase of Toll-like receptor 4 immunoreactive (TLR4 + IR) cells in the adult basal forebrain of AIE-treated subjects, relative to CONs. Adolescent galantamine treatment alone from P25 to P54 decreased constitutive expression of TLR4 + IR cells in CONs and prevented the AIE-induced increase of TLR4 + IR cells in adulthood, relative to vehicle-treated AIE subjects. **(C)** Modified unbiased stereological assessment revealed a 50% (±9%) increase of receptor for advanced glycation end-products immunoreactive (RAGE + IR) cells in the adult basal forebrain of AIE-treated subjects, relative to CONs. Adolescent galantamine treatment from P25 to P54 did not affect RAGE + IR in CONs, but prevented the AIE-induced increase of RAGE + IR cells in adulthood, relative to vehicle-treated AIE subjects. **(D)** Modified unbiased stereological assessment revealed an 18% (±2%) increase of phosphorylated nuclear factor kappa-light-chain-enhancer of activated B cells p65 immunoreactive (pNF-κB p65 + IR) cells in the adult basal forebrain of AIE-treated subjects, relative to CONs. Adolescent galantamine treatment alone from P25 to P54 decreased constitutive expression of pNF-κB p65 + IR cells in CONs and prevented the AIE-induced increase of pNF-κB p65 + IR cells in adulthood, relative to vehicle-treated AIE subjects. **(E)** Immunofluorescent co-labeling analysis revealed a 95% (±17%) increase of choline acetyltransferase immunoreactive (ChAT + IR) neurons that co-expressed pNF-κB p65 in the adult basal forebrain of AIE-treated subjects, relative to CONs. Galantamine treatment alone did not affect ChAT colocalization with pNF-κB p65 in CONs, but prevented the AIE-induced increase of ChAT colocalization with pNF-κB p65 in the adult basal forebrain, relative to vehicle-treated AIE subjects. Representative fluorescent photomicrographs of ChAT (green) and pNF-κB p65 (red) colocalization in the adult basal forebrain of CON- and AIE-treated subjects. Closed arrowheads = ChAT + IR neuron colocalization with pNF-κB p65 + (yellow); open arrowheads = ChAT + IR neurons that did not co-express pNF-κB p65. Scale bar = 50 μm. Data are presented as mean ± SEM (n = 8/group). ^∗^*p* < 0.05, ^∗∗^*p* < 0.01.

Emerging studies find HMGB1 release contributes to induction of TLR and RAGE neuroimmune receptors as well as other neuroimmune genes, contributing to neurodegeneration in brain diseases, including AD and AUD (Crews et al., 2013; Vetreno et al., 2013, 2018; Heneka et al., 2015; Paudel et al., 2020). We find AIE causes persistent upregulation of HMGB1, RAGE, TLR4, NF-κB, and other neuroimmune signaling molecules in brain with associated regional adult neuropathology (Vetreno and Crews, 2012, 2018; Vetreno et al., 2013, 2018, 2019). We assessed whether AIE combined with galantamine, which is reported to exert anti-inflammatory effects (Liu et al., 2018), would prevent the lasting activation of proinflammatory neuroimmune signaling in the adult basal forebrain. We find an AIE-induced 48% (±7%) increase of HMGB1 + IR in the adult basal forebrain [one-way ANOVA: *F*_(__1_, _14__)_ = 24.4, *p* < 0.01; see Figure 3A], relative to CON subjects. While adolescent galantamine treatment alone did not affect HMGB1+, it blunted the AIE-induced increase of HMGB1 + IR cells in the adult basal forebrain. HMGB1 is an endogenous agonist at TLR4, RAGE, and other proinflammatory neuroimmune receptors (Andersson et al., 2018; Paudel et al., 2020). We report a 14% (±2%) increase of TLR4 + IR cells (Tukey’s HSD: *p* < 0.05; see Figure 3B) and a 50% (±9%) increase of RAGE + IR cells (Tukey’s HSD: *p* < 0.01; see Figure 3C) in the basal forebrain of adult AIE-treated rats, relative to CON subjects. Adolescent galantamine treatment alone decreased constitutive expression of TLR4 + (Tukey’s HSD: *p* < 0.05) but not RAGE+, and prevented the AIE-induced increase of TLR4 + IR (Tukey’s HSD: *p* < 0.01) and RAGE + IR cells (Tukey’s HSD: *p* < 0.01) in the adult basal forebrain, relative to vehicle-treated AIE subjects. Thus, galantamine, a cholinesterase inhibitor that increases acetylcholine, blocks the AIE-induced increases of proinflammatory HMGB1, TLR4, and RAGE expression as well as somal shrinkage and loss of BFCNs.

HMGB1 activation of TLR4 and RAGE leads to downstream activation of NF-κB (Chavakis et al., 2004; Tobon-Velasco et al., 2014; Liu et al., 2017; Aucott et al., 2018). NF-κB is a nuclear transcription factor known to induce multiple neuroimmune genes (Pahl, 1999), and activated pNF-κB p65 provides insight into neuroimmune signaling in brain (Vetreno et al., 2017, 2018; Vetreno and Crews, 2018). We report a significant AIE-induced 18% (±2%) increase of pNF-κB p65 + IR cells in the adult basal forebrain (Tukey’s HSD: *p* < 0.01), relative to CON subjects. Galantamine treatment alone during adolescence decreased constitutive expression of pNF-κB p65 + in CONs (Tukey’s HSD: *p* < 0.01) and prevented the AIE-induced increase of pNF-κB p65+, relative to vehicle-treated AIE subjects (Tukey’s HSD: *p* < 0.01; see Figure 3D). Emerging studies suggest both glia and neurons are involved in brain neuroimmune signaling (Crews et al., 2013, 2017a; Erickson et al., 2019), prompting a determination of co-localization of pNF-κB p65 + with ChAT + IR BFCNs. Interestingly, AIE increased ChAT + /pNF-κB p65 + colocalized cells by approximately twofold (Tukey’s HSD: *p* < 0.01) in the adult AIE-treated basal forebrain, relative to CON subjects (see Figure 3E). These data suggest that direct neuroimmune signaling within BFCNs is increased by AIE. Adolescent galantamine treatment alone did not affect ChAT + /pNF-κB p65 + colocalization in CONs, but galantamine treatment combined with AIE prevented the AIE-induced increase of ChAT + /pNF-κB p65 + colocalization in the adult basal forebrain (Tukey’s HSD: *p* < 0.01), relative to vehicle-treated AIE subjects. Together, these data suggest that the AIE-induced increase of HMGB1-TLR4/RAGE-pNF-κB p65 expression, including increased pNF-κB p65 colocalization within ChAT + BFCNs, contributes to the loss of ChAT, TrkA, and p75^NTR^ expression. Further, these findings are consistent with galantamine increasing anti-inflammatory acetylcholine by blocking cholinesterase, which prevents increases of HMGB1-TLR4/RAGE-pNF-κB p65 signaling and the concomitant loss and somal shrinkage of BFCNs.

### Adolescent Galantamine Treatment During AIE Prevents the AIE-Induced Increase of Epigenetic Histone Silencing Markers in the Adult Basal Forebrain

**FIGURE 4:**
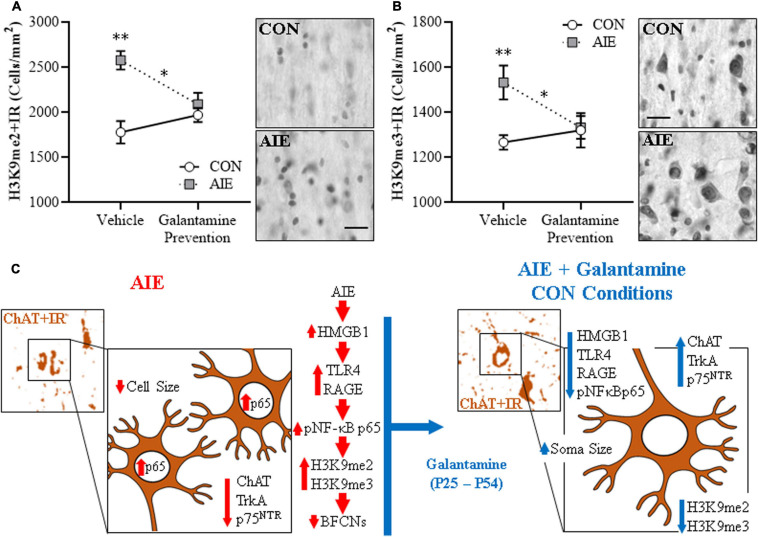
Adolescent galantamine treatment prevented the adolescent intermittent ethanol (AIE)-induced increase of histone methylation markers in the adult basal forebrain. **(A)** Modified unbiased stereological assessment revealed a 45% (± 6%) increase of histone 3 lysine 9 dimethylation immunoreactive (H3K9me2 + IR) cells in the adult (P70) basal forebrain of AIE-treated subjects, relative to CONs. Adolescent galantamine treatment alone from P25 to P54 did not affect expression of H3K9me2 + IR cells in CONs, but prevented the AIE-induced increase of H3K9me2 + IR cells in adulthood (P70), relative to vehicle-treated AIE subjects. Representative photomicrographs of H3K9me2 + IR cells in the adult basal forebrain of CON- and AIE-treated subjects. **(B)** Modified unbiased stereological assessment revealed a 21% (±6%) increase of histone 3 lysine 9 trimethylation immunoreactive (H3K9me3 + IR) cells in the adult basal forebrain of AIE-treated subjects, relative to CONs. Adolescent galantamine treatment alone from P25 to P54 did not affect expression of H3K9me3 + IR cells in CONs, but prevented the AIE-induced increase of H3K9me3 + IR cells in adulthood, relative to vehicle-treated AIE subjects. Representative photomicrographs of H3K9me3 + IR cells in the adult basal forebrain of CON- and AIE-treated subjects. Scale bar = 50 μm. Data are presented as mean ± SEM (*n* = 8/group). ^∗^*p* < 0.05, ^∗∗^*p* < 0.01. **(C)** Simplified schematic depicting the proposed neuroimmune and epigenetic mechanism underlying the persistent AIE-induced loss of basal forebrain cholinergic neurons (BFCNs). (Left) AIE treatment from P25 to P54 causes the loss of BFCN markers and somal shrinkage of the residual cholinergic neurons in the adult (P70) basal forebrain. (Small panel). Representative photomicrograph of ChAT + IR neurons in the adult (P70) AIE-treated basal forebrain. (Large panel) Schematic depicting AIE-induced decreased expression of BFCN markers (i.e., ChAT, TrkA, p75^NTR^), somal shrinkage of the residual ChAT + neurons, and increased expression of activated pNF-κB p65 within ChAT + BFCNs. In the proposed mechanism, AIE treatment increases expression of the endogenous proinflammatory TLR4/RAGE ligand HMGB1 and the HMGB1 receptors TLR4 and RAGE leading to increased BFCN expression of activated pNF-κB p65 and increased expression of the histone 3 lysine 9 (H3K9) methylation markers (i.e., dimethylation and trimethylation) resulting in loss of BFCN markers. (Right) Galantamine treatment combined with AIE prevents the AIE-induced loss of BFCN markers and somal shrinkage of the residual cholinergic neurons in the adult basal forebrain. (Small panel) Representative photomicrograph of ChAT + IR neurons in the adult AIE-treated basal forebrain following adolescent galantamine treatment. (Large panel) Adolescent galantamine treatment (i.e., P25–P54) combined with AIE blocks the induction of HMGB1-TLR4/RAGE-pNF-κB p65 signaling, increase of histone 3 methylation markers, and provides long-lasting recovery of the somal shrinkage and loss of BFCN markers in the adult basal forebrain.

Emerging studies find epigenetic mechanisms are related to neuroimmune gene induction and persistent AIE pathology (Montesinos et al., 2016; Pandey et al., 2017; Crews et al., 2019). We previously found AIE-induced increases of histone 3 lysine 9 dimethylation (H3K9me2) occupancy at *ChAT* and *TrkA* gene promoters, which is known to silence gene transcription, that appears to contribute to the loss of ChAT + neurons (Wang et al., 2008; Vetreno et al., 2019). In the present experiment, we find AIE induced a 45% (±6%) increase of H3K9me2 + IR cells and a 21% (±6%) increase of H3K9me3 + IR cells (Tukey’s HSD: *p* < 0.01) in the adult basal forebrain, relative to CON subjects (see Figures 4A,B). Adolescent galantamine treatment alone did not affect expression of these epigenetic gene silencing markers in CONs, but AIE combined with galantamine prevented the AIE-induced increases of H3K9me2 + IR (Tukey’s HSD: *p* < 0.05) and H3K9me3 + IR (Tukey’s HSD: *p* < 0.05) in the adult basal forebrain, relative to vehicle-treated AIE subjects. We did not observe an effect of AIE or galantamine treatment on expression of acetylated H3K9 (H3K9ac + IR cells: CON/Vehicle: 5,186 ± 124, CON/Galantamine: 5,112 ± 73, AIE/Vehicle: 5,153 ± 138, AIE/Galantamine: 5,136 ± 110). Taken together, these experiments suggest that blockade of AIE induction of HMGB1-TLR4/RAGE-pNF-κB p65 signaling and increases in H3K9me2 + and H3K9me3 + by adolescent galantamine treatment prevents the somal shrinkage and loss of BFCN markers in the adult basal forebrain (see Figure 4C). These studies are consistent with AIE-induced ChAT + neuron loss involving epigenetic gene silencing mechanisms that were initially thought to reflect cell death.

### Galantamine Treatment Post-AIE Restores Cholinergic Neuron Markers and Somal Shrinkage of Residual ChAT + IR Neurons in the Adult Basal Forebrain

**FIGURE 5:**
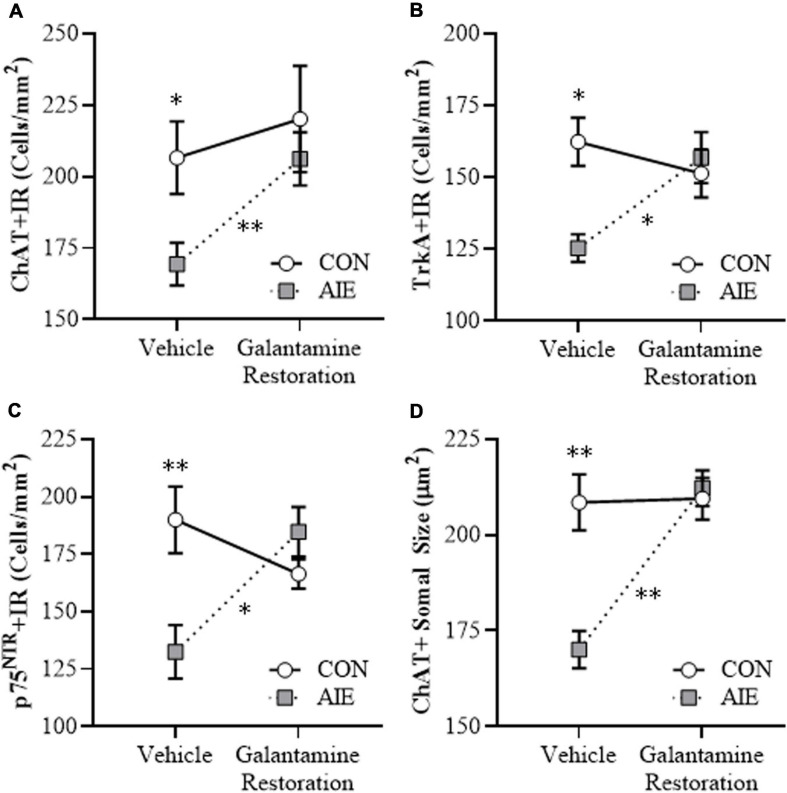
Adult galantamine treatment restored the adolescent intermittent ethanol (AIE)-induced loss and somal shrinkage of cholinergic neurons in the adult basal forebrain. **(A)** Modified unbiased stereological assessment revealed an 18% (±4%) reduction of choline acetyltransferase immunoreactive (ChAT + IR) neurons in the adult (P73) basal forebrain of AIE-treated subjects, relative to CONs. Adult galantamine treatment alone from P57 to P73 did not affect ChAT + IR in CONs, but restored the AIE-induced loss of ChAT + IR neurons in adulthood, relative to vehicle-treated AIE subjects. **(B)** Modified unbiased stereological assessment revealed a 23% (±3%) reduction of tropomyosin receptor kinase A immunoreactive (TrkA + IR) neurons in the adult (P73) basal forebrain of AIE-treated subjects, relative to CONs. Adult galantamine treatment alone did not affect TrkA + IR in CONs, but restored the AIE-induced loss of TrkA + IR neurons in adulthood, relative to vehicle-treated AIE subjects. **(C)** Modified unbiased stereological assessment revealed a 30% (±6%) reduction of p75 neurotrophin receptor immunoreactive (p75^NTR^ + IR) neurons in the adult (P73) basal forebrain of AIE-treated subjects, relative to CONs. Adult galantamine treatment alone did not affect p75^NTR^ + IR in CONs, but restored the AIE-induced loss of p75^NTR^ + IR neurons in adulthood, relative to vehicle-treated AIE subjects. **(D)** Analysis of ChAT + neuron somal size revealed an 18% (±2%) reduction in somal size of the residual ChAT + neurons in the adult basal forebrain of AIE-treated subjects, relative to CONs. Adult galantamine treatment alone did not affect ChAT + somal size, but restored the AIE-induced somal shrinkage of ChAT + cholinergic neurons in the adult basal forebrain, relative to vehicle-treated AIE subjects. Data are presented as mean ± SEM (n = 8/group). ^∗^*p* < 0.05, ^∗∗^*p* < 0.01.

Emerging studies find that persistent AIE-induced adult pathology includes complex epigenetic mechanisms that underlie lasting changes in adult gene expression, hippocampal neurogenesis, ethanol drinking and preference, anxiety, cognitive deficits, and reduced behavioral flexibility (Pandey et al., 2015; Sakharkar et al., 2016; Mulholland et al., 2018; Kyzar et al., 2019; Vetreno et al., 2019). Interestingly, anti-inflammatory indomethacin, the cholinesterase inhibitor donepezil, and the histone deacetylase inhibitor TSA have been found to reverse adult AIE-induced brain cellular and behavioral pathology (Crews et al., 2019). We sought to determine if adult treatment with galantamine following the conclusion of AIE (i.e., P57–P72) would restore the loss of BFCN markers in the adult (i.e., P73) basal forebrain. In this replicate, we included an additional group of AIE subjects that we sacrificed at P57 and find AIE caused a 22% (±5%) reduction of ChAT + IR neurons (*t*_[__14__]_ = 3.3, *p* < 0.01; ChAT + IR cells: CON: 208 ± 9, AIE: 162 ± 11) in the late adolescent basal forebrain, relative to age-matched CON subjects. In the adult (i.e., P73) basal forebrain following AIE treatment, we find a persistent AIE-induced 18% (± 4%) reduction of ChAT + IR cells [one-way ANOVA: *F*_(__1_, _14__)_ = 6.4, *p* < 0.05], a 23% (±3%) reduction of TrkA + IR cells (Tukey’s HSD: *p* < 0.05), and a 30% (± 6%) reduction of p75^NTR^ + IR cells (Tukey’s HSD: *p* < 0.01), relative to age-matched CON subjects (see Figures 5A–C). Adult galantamine treatment alone did not affect expression of BFCN markers in CONs but restored the AIE-induced loss of ChAT + IR cells [one-way ANOVA: *F*_(__1_, _14__)_ = 9.6, *p* < 0.01], TrkA + IR cells (Tukey’s HSD: *p* < 0.05), and p75^NTR^ + IR cells (Tukey’s HSD: *p* < 0.05) in the adult basal forebrain, relative to vehicle-treated AIE subjects. Similarly, we report a significant 18% (±2%) reduction in somal size of the residual ChAT + neurons in the adult basal forebrain (Tukey’s HSD: *p* < 0.01), relative to CON subjects. Galantamine treatment alone did not affect ChAT + somal size, but reversed the AIE-induced somal shrinkage of ChAT + BFCNs in the adult basal forebrain (Tukey’s HSD: *p* < 0.01; see Figure 5D), relative to vehicle-treated AIE subjects. Thus, AIE caused a loss of ChAT + BFCNs in the late adolescent (i.e., P57) basal forebrain that persisted into adulthood (i.e., P73), and post-AIE galantamine treatment reversed the AIE-induced loss of BFCN markers and somal shrinkage of the residual ChAT + neurons in the adult basal forebrain.

### Galantamine Reverses the AIE-Induced Increase of Proinflammatory Neuroimmune Signaling Molecules in the Adult Basal Forebrain

**FIGURE 6:**
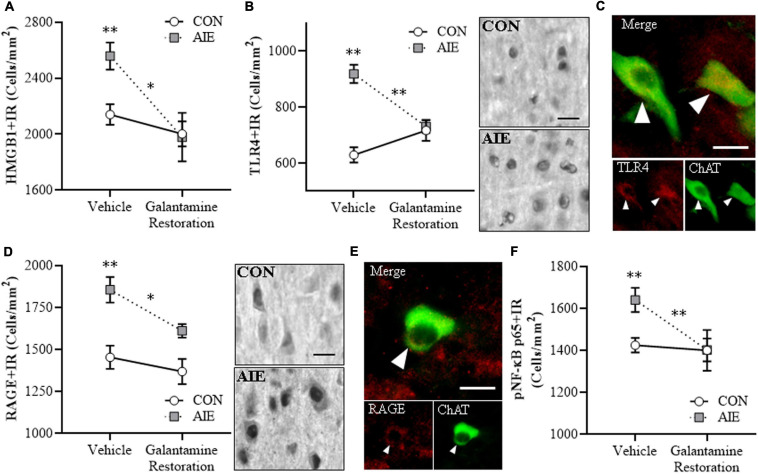
Adult galantamine treatment reversed the adolescent intermittent ethanol (AIE)-induced increase of proinflammatory neuroimmune signaling molecules in the adult basal forebrain. **(A)** Modified unbiased stereological assessment revealed a 20% (±5%) increase of high-mobility group box 1 immunoreactive (HMGB1 + IR) cells in the adult (P73) basal forebrain of AIE-treated subjects, relative to CONs. Adult galantamine treatment from P57 to P73 did not affect HMGB1 + IR in CONs, but reversed the AIE-induced increase of HMGB1 + IR cells in adulthood, relative to vehicle-treated AIE subjects. **(B)** Modified unbiased stereological assessment revealed a 46% (±5%) increase of Toll-like receptor 4 immunoreactive (TLR4 + IR) cells in the adult (P73) basal forebrain of AIE-treated subjects, relative to CONs. Adult galantamine treatment alone did not affect expression of TLR4 + IR cells in CONs, but reversed the AIE-induced increase of TLR4 + IR cells in adulthood, relative to vehicle-treated AIE subjects. Representative photomicrographs of TLR4 + IR cells in the adult basal forebrain of CON- and AIE-treated subjects. Scale bar = 50 μm. **(C)** Immunofluorescent co-labeling revealed TLR4 (red) colocalization with choline acetyltransferase immunoreactive (ChAT + IR) neurons in the adult basal forebrain. Closed arrowheads = TLR4 + IR colocalization with ChAT + IR neurons (yellow). Scale bar = 50 μm. **(D)** Modified unbiased stereological assessment revealed a 28% (±5%) increase of receptor for advanced glycation end-products immunoreactive (RAGE + IR) cells in the adult (P73) basal forebrain of AIE-treated subjects, relative to CONs. Adult galantamine treatment alone did not affect RAGE + IR in CONs, but reversed the AIE-induced increase of RAGE + IR cells in adulthood, relative to vehicle-treated AIE subjects. Representative photomicrographs of RAGE + IR cells in the adult basal forebrain of CON- and AIE-treated subjects. Scale bar = 50 μm. **(E)** Immunofluorescent co-labeling revealed RAGE (red) colocalization with ChAT + IR neurons in the adult basal forebrain. Closed arrowheads = RAGE + IR colocalization with ChAT + IR neurons (yellow). Scale bar = 50 μm. **(F)** Modified unbiased stereological assessment revealed a 15% (±4%) increase of phosphorylated nuclear factor kappa-light-chain-enhancer of activated B cells p65 immunoreactive (pNF-κB p65 + IR) cells in the adult (P73) basal forebrain of AIE-treated subjects, relative to CONs. Adult galantamine treatment alone did not affect pNF-κB p65 + IR cells in CONs, but reversed the AIE-induced increase of pNF-κB p65 + IR cells in adulthood, relative to vehicle-treated AIE subjects. Data are presented as mean ± SEM (*n* = 8/group). ^∗^*p* < 0.05, ^∗∗^*p* < 0.01.

The post-AIE galantamine reversal of AIE-induced reductions of BFCN markers prompted determination of neuroimmune HMGB1-TLR4/RAGE-pNF-κB p65 signal expression in the adult (i.e., P73) basal forebrain. Consistent with our earlier studies, AIE treatment caused a 20% (± 5%) increase of HMGB1 + IR cells [one-way ANOVA: *F*_(__1_, _14__)_ = 11.7, *p* < 0.01], a 46% (±5%) increase of TLR4 + IR cells (Tukey’s HSD: *p* < 0.01), a 28% (±5%) increase of RAGE + IR cells [one-way ANOVA: *F*_(__1_, _14__)_ = 15.2, *p* < 0.01], and a 15% (±4%) increase of pNF-κB p65 + IR cells [one-way ANOVA: *F*_(__1_, _14__)_ = 10.3, *p* < 0.01] in the adult basal forebrain, relative to CON subjects (see Figure 6). Adult galantamine treatment alone did not affect expression of proinflammatory neuroimmune markers in CONs, but post-AIE treatment with galantamine reversed the AIE-induced increase of HMGB1 + [one-way ANOVA: *F*_(__1_, _14__)_ = 8.5, *p* < 0.05], TLR4 + (Tukey’s HSD: *p* < 0.01), RAGE + [one-way ANOVA: *F*_(__1_, _14__)_ = 8.0, *p* < 0.05], and pNF-κB p65 + [one-way ANOVA: *F*_(__1_, _14__)_ = 9.1, *p* < 0.01] cells in the adult basal forebrain, relative to vehicle-treated AIE subjects. Double immunofluorescent IHC for ChAT with TLR4 + and RAGE + suggests that some of the increases in signaling occur within ChAT + BFCNs (see Figures 6C,E). Thus, treatment with galantamine following AIE-induced pathology reversed the AIE-induced increase of HMGB1-TLR4/RAGE-pNF-κB p65 signal expression in the adult basal forebrain that paralleled the restoration of BFCNs.

### Galantamine Reverses the AIE-Induced Increases of Cellular and Cholinergic Promoter Histone Silencing Markers in the Adult Basal Forebrain

We further tested our hypothesis that AIE increases of HMGB1-TLR4/RAGE-pNF-κB p65 signaling within BFCNs contribute to silencing of cholinergic phenotype genes by determining if post-AIE galantamine treatment reverses the AIE-induced increase of H3K9 methylation markers.

**FIGURE 7:**
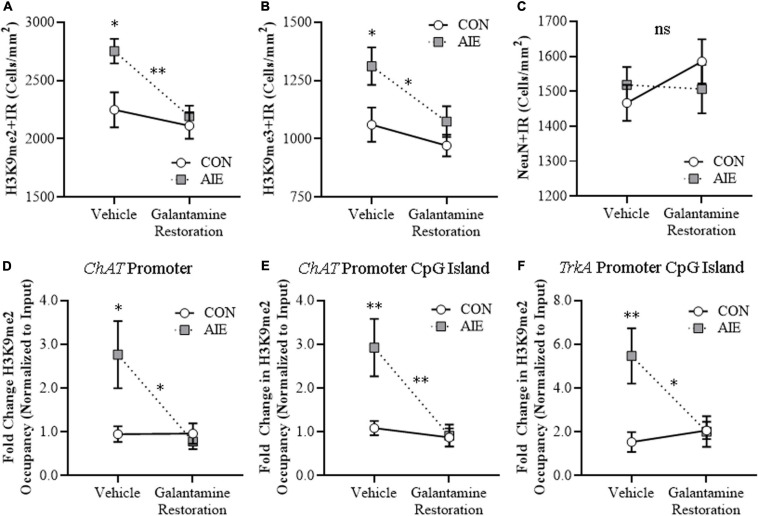
Adult galantamine treatment reversed the adolescent intermittent ethanol (AIE)-induced increase of histone methylation markers and cholinergic gene histone methylation in the adult basal forebrain. **(A)** Modified unbiased stereological assessment revealed a 22% (±5%) increase of histone 3 lysine 9 dimethylation immunoreactive (H3K9me2 + IR) cells in the adult (P73) basal forebrain of AIE-treated subjects, relative to CONs. Adult galantamine treatment alone from P57 to P73 did not affect expression of H3K9me2 + IR cells in CONs, but reversed the AIE-induced increase of H3K9me2 + IR cells in adulthood, relative to vehicle-treated AIE subjects. **(B)** Modified unbiased stereological assessment revealed a 24% (±8%) increase of histone 3 lysine 9 trimethylation immunoreactive (H3K9me3 + IR) cells in the adult basal forebrain of AIE-treated subjects, relative to CONs. Adult galantamine treatment alone did not affect expression of H3K9me3 + IR cells in CONs, but reversed the AIE-induced increase of H3K9me3 + IR cells in adulthood, relative to vehicle-treated AIE subjects. **(C)** Modified unbiased stereological assessment of the neuronal marker NeuN in the adult basal forebrain revealed that neither AIE or galantamine treatment affected NeuN + IR neuron counts, relative to CONs. **(D)** Chromatin immunoprecipitation (ChIP) assessment revealed that occupancy of H3K9me2 at the promoter of the *ChAT* gene increased by approximately 1.9-fold in the basal forebrain of adult (P73) AIE-treated animals, relative to CONs. Adult galantamine treatment from P57 to P73 did not affect levels of H3K9me2 in CONs, but resolved the AIE-induced increase of H3K9me2 at the promoter of the *ChAT* gene. **(E)** ChIP assessment revealed that levels of H3K9me2 at the CpG island in the *ChAT* promoter were increased by approximately 1.7-fold in the basal forebrain of adult AIE-treated animals, relative to CONs. Adult galantamine treatment alone did not affect levels of H3K9me2 in CONs, but resolved the AIE-induced increase of H3K9me2 at the CpG island in the *ChAT* promoter. **(F)** ChIP assessment revealed that levels of H3K9me2 at the CpG island in the *TrkA* promoter were increased by approximately 2.6-fold in the basal forebrain of adult AIE-treated animals, relative to CONs. Adult galantamine treatment alone did not affect levels of H3K9me2 in CONs, but resolved the AIE-induced increase of H3K9me2 at the CpG island in the *TrkA* promoter. Data are presented as mean ± SEM (*n* = 8/group). ns = non-significant, ^∗^*p* < 0.05, ^∗∗^*p* < 0.01.

We find an AIE increase of H3K9me2 + IR cells [22% (±5%), one-way ANOVA: *F*_(__1_, _14__)_ = 7.5, *p* < 0.05] and H3K9me3 + IR cells [24% (±8%), one-way ANOVA: *F*_(__1_, _14__)_ = 5.4, *p* < 0.05] in the adult basal forebrain, relative to CON subjects (see Figures 7A,B). While adult galantamine treatment alone did not affect expression of H3K9 methylation markers in CONs, post-AIE galantamine treatment reversed the AIE-induced increase of H3K9me2 + IR cells [one-way ANOVA: *F*_(__1_, _14__)_ = 16.0, *p* < 0.01] and H3K9me3 + IR cells [one-way ANOVA: *F*_(__1_, _14__)_ = 5.3, *p* < 0.05], relative to vehicle-treated AIE subjects. Although BFCN markers decreased and epigenetic silencing markers increased following AIE treatment, there were no changes in neuronal density across treatment conditions as determined by expression of the neuronal marker NeuN (see Figure 7C), consistent with our earlier studies (Vetreno et al., 2019). To determine cholinergic gene-specific information, we assessed histone methylation silencing within *ChAT* and *TrkA* gene promoters, which are two cholinergic phenotype genes. We report that AIE treatment increased levels of H3K9me2 occupancy by approximately 1.9-fold at the *ChAT* promoter (Tukey’s HSD: *p* < 0.05), 1.7-fold at the CpG island of the *ChAT* promoter (Tukey’s HSD: *p* < 0.01), and 2.6-fold at the CpG island of the *TrkA* promoter (Tukey’s HSD: *p* < 0.01) in the adult basal forebrain, relative to CON subjects (see Figures 7D–F). While adult galantamine treatment alone did not affect levels of H3K9me2 occupancy in CONs, it reversed the AIE-induced increase of H3K9me2 occupancy at the *ChAT* promoter (Tukey’s HSD: *p* < 0.05), the CpG island of the *ChAT* promoter (Tukey’s HSD: *p* < 0.01), and the CpG island of the *TrkA* promoter (Tukey’s HSD: *p* < 0.05), relative to vehicle-treated AIE subjects. Levels of H3K9me2 were unchanged at the TrkA promoter (data not shown). The loss of BFCN markers along with increases of H3K9me2 at both *ChAT* and *TrkA* promoters, but no change in total neurons, supports epigenetic programming leading to long-lasting silencing of the cholinergic phenotype.

## Discussion

**FIGURE 8:**
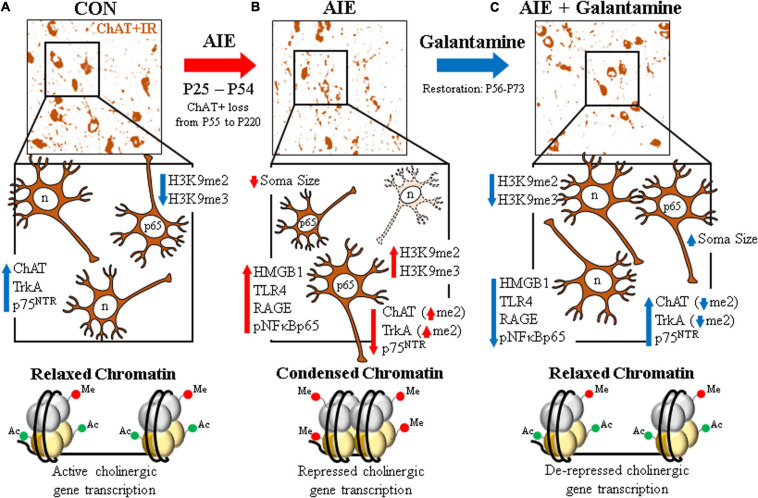
Schematic depicting the proposed mechanism underlying the persistent adolescent intermittent ethanol (AIE)-induced loss of basal forebrain cholinergic neurons. **(A)** In the naïve basal forebrain, cholinergic neurons express choline acetyltransferase (ChAT), the high-affinity nerve growth factor (NGF) receptor tropomyosin receptor kinase A (TrkA), and the low-affinity NGF receptor p75 neurotrophin receptor (p75^NTR^) (Vetreno et al., 2019). (Top) ChAT + IR neurons in the CON-treated adult (P73) basal forebrain. (Middle) Schematic depicting ChAT + IR basal forebrain cholinergic neurons in orange. Note that “n” in the nucleus represents neuronal NeuN immunoreactivity. Constitutive expression of proinflammatory signaling molecules and histone methylation markers are relatively low in the naïve basal forebrain. (Bottom) Schematic depicting relaxed, open chromatin allowing active transcription of cholinergic genes and maintenance of the cholinergic phenotype. **(B)** AIE causes the loss of ChAT + IR neurons in the adolescent (P55) basal forebrain that persists into adulthood (P200) (Vetreno et al., 2014). (Top) ChAT + IR neuron loss and shrinkage of the residual cholinergic neurons in the adult (P73) AIE-treated basal forebrain. (Middle) AIE-induced loss of ChAT-, TrkA-, and p75^NTR^-immunoreactive basal forebrain neurons (dashed neuron lacking ChAT [orange]) as well as somal shrinking of the residual ChAT + IR cholinergic neurons. AIE increased expression of the endogenous ligand HMGB1, the HMGB1 receptors TLR4 and RAGE, and phosphorylation of the proinflammatory transcription factor NF-κB p65 in the adult basal forebrain, and neuroimmune signaling might alter gene expression in part through epigenetic mechanisms (Montesinos et al., 2016). Note that “n” in the nucleus represents NeuN + IR, which was unchanged by AIE, consistent with the loss of the cholinergic phenotype and not cell death (Vetreno et al., 2019). AIE increased expression of histone 3 methylation markers (i.e., H3K9me2 and H3K9me3) and dimethylation of lysine 9 of histone 3 (H3K9me2) associated with promoter regions on the *ChAT* and *TrkA* genes in the adult basal forebrain. (Bottom) Schematic depicting condensed, closed chromatin repressing transcription of cholinergic genes. **(C)** Galantamine treatment from P57 to P72 restored the AIE-induced loss of basal forebrain cholinergic neurons. (Top) Galantamine restoration of the AIE-induced loss of ChAT + IR neurons and somal shrinkage of the residual cholinergic neurons in the adult (P73) basal forebrain. (Middle) Restoration of ChAT-, TrkA, and p75^NTR^-immunoreactive basal forebrain neurons, and reversal of cholinergic neuron shrinkage in the adult basal forebrain of AIE-treated subjects. Restorative galantamine treatment reversed the AIE-induced increased expression of proinflammatory neuroimmune signaling molecules (i.e., HMGB1, RAGE/TLR4, and pNF-κB p65), histone methylation markers (i.e., H3K9me2 and H3K9me3), and increase of H3K9me2 in promoter regions of both the *ChAT* and *TrkA* genes in the adult basal forebrain. These data suggest that galantamine is a potential treatment modality for maladaptive changes in neural architecture in the adult basal forebrain that may recover cognitive deficits associated with cholinergic system dysfunction. (Bottom) Schematic depicting galantamine restoration of condensed chromatin to relaxed, open chromatin state allowing de-repression and active transcription of cholinergic genes and restoration of the cholinergic neuron phenotype.

The current study tested the hypothesis that treatment with the cholinesterase inhibitor galantamine, which has FDA approval for the treatment of AD (Lilienfeld, 2002; Hampel et al., 2018; Haake et al., 2020), would recover the lasting adult basal forebrain cholinergic pathology associated with adolescent binge ethanol exposure. Degeneration of BFCNs and ChAT + somal shrinkage is a feature of several neurodegenerative disorders, including AD and AUD (Lehericy et al., 1993; Vetreno et al., 2014), and may contribute to the cognitive deficits associated with these disorders (Vogels et al., 1990; De Rosa et al., 2004; Mufson et al., 2008). Galantamine treatment during AIE (i.e., P25–P54) or following the conclusion of AIE (i.e., P57–P72) recovered the persistent loss of cholinergic neuron markers (i.e., ChAT, TrkA, and p75^NTR^) and somal shrinkage of residual BFCNs in the adult basal forebrain. However, similar to previous findings (Vetreno et al., 2019), we found no loss of NeuN + IR, a global neuronal marker, consistent with no loss of neurons, only cholinergic phenotype markers. We and others (Pascual et al., 2007; Vetreno and Crews, 2018; Vetreno et al., 2018) have previously found the anti-inflammatory drug indomethacin can prevent ethanol induction of brain proinflammatory neuroimmune genes and neuropathology, including loss of ChAT + IR neurons, consistent with loss of BFCNs being mediated by proinflammatory neuroimmune and epigenetic gene silencing histone (H3K9) methylation mechanisms. We found galantamine treatment during AIE or initiated 72 h post-AIE blocked the AIE-induced increased expression of the proinflammatory neuroimmune receptors TLR4 and RAGE, the endogenous TLR4/RAGE ligand HMGB1, and activation of pNF-κB p65 in the adult basal forebrain. Further, we found BFCNs express TLR4, RAGE, and activated pNF-κB p65, and that AIE treatment increased pNF-κB p65 expression in adult ChAT + IR neurons that was reversed by galantamine treatment consistent with galantamine inhibiting neuroimmune signaling within BFCNs. Previous studies found AIE increased HMGB1, TLRs, RAGE, pNF-κB p65, and other neuroimmune genes that persist into adulthood across multiple brain regions, including the forebrain, cortex, hippocampus, and cerebellum (Vetreno and Crews, 2012; Vetreno et al., 2013, 2018, 2019; Crews et al., 2017b). We previously reported that exercise during or following AIE treatment can restore ChAT +, TrkA +, and p75^NTR^ + loss as well as associated adult cognitive deficits (Vetreno et al., 2019). In the present study, we report that galantamine treatment recovered the AIE-induced increase of cellular histone 3 methylation markers (i.e., H3K9me2 and H3K9me3) as well as increased H3K9me2 occupancy at *ChAT* and *TrkA* gene promoters. Together, these data support the hypothesis that AIE increases HMGB1+, TLR4+, and RAGE+, and downstream pNF-κB p65 expression within ChAT + BFCNs. As shown in Figure 8, relaxed chromatin within cholinergic phenotype genes allow high levels of expression in the healthy adult brain that are reduced by AIE along with ChAT + cholinergic neuron shrinkage. The increases in HMGB1, TLR4, RAGE, and pNF-κB p65 are associated with increases in both cellular histone 3 silencing markers and cholinergic gene promoter methylation, consistent with AIE inducing condensed chromatin that decreases expression of cholinergic phenotype genes, causes shrinkage and loss of BFCNs. Galantamine combined with or following AIE reverses increases of HMGB1, TLR4, RAGE, pNF-κB p65, and histone 3 silencing markers, opening chromatin and restoring gene expression and BFCN markers. Neuroimmune-linked epigenetic alterations in neuronal phenotype may represent a previously unappreciated mechanism of plasticity. Galantamine restoration of the AIE-induced persistent loss of BFCNs through reversal of persistent neuroimmune-induced changes in chromatin epigenetic silencing may offer great promise for reversing AIE pathology as well as other pathologies initially thought to be irreversible.

Galantamine is an herbal alkaloid that inhibits cholinesterase and is used to treat cholinergic hypofunction in AD. By inhibiting cholinesterase, galantamine increases acetylcholine bioavailability, which is known to block induction of proinflammatory genes. While the mechanisms underlying the loss of BFCNs remain to be fully elucidated, accumulating evidence implicates activation of the proinflammatory neuroimmune signaling system (Vetreno and Crews, 2018). HMGB1-RAGE/TLR4-NF-κB p65 neuroimmune signaling has been implicated in neuropathology associated with AD and AUD (Boissiere et al., 1997; Crews et al., 2013; Vetreno et al., 2013; Fujita et al., 2016; Paudel et al., 2020). The anti-inflammatory effects of galantamine have been reported in rodent models of endotoxemia, obesity, colitis, and other disorders (Pavlov et al., 2009; Satapathy et al., 2011; Wazea et al., 2018). Acetylcholine is anti-inflammatory as ACh suppresses LPS-induced release of HMGB1 and nicotinic ACh receptor (nAChR) activation prevents activity of the NF-κB signaling pathway (Wang et al., 2004). Galantamine is not only a cholinesterase inhibitor, but also acts as a positive allosteric ligand at nAChRs, including α7 nAChRs (Wazea et al., 2018), and potentiates cholinergic transmission by positively modulating the response of nAChR to ACh and their agonists (Dajas-Bailador et al., 2003; Samochocki et al., 2003). α7 nAChRs are present on microglia and neurons, including BFCNs (Azam et al., 2003; Ulloa, 2005), and play a role in modulating neuroinflammation (Sitapara et al., 2014; Ren et al., 2017; Wazea et al., 2018). Alternatively, galantamine may exert neuroprotective effects on BFCNs through interactions with the high-affinity NGF receptor TrkA. Studies suggest that the AIE-induced loss of trophic factor receptors, such as the high affinity NGF receptor TrkA, might contribute to the loss of ChAT + IR neurons. NGF and TrkA are critical for the survival and maintenance of BFCNs (Hagg et al., 1988, 1989; Fagan et al., 1997; Kim et al., 2014; Allaway and Machold, 2017), and galantamine has been reported to activate TrkA receptors, leading to phosphorylation of the transcription factor CREB (Autio et al., 2011). While the precise mechanism underlying the galantamine reversal of HMGB1-TLR4/RAGE-pNF-κB p65 neuroimmune signaling remains to be fully elucidated, our findings that galantamine is capable of recovering the persistent loss of BFCNs and lasting activation of neuroimmune and gene silencing have broad implications that could impact AUD as well as AD.

Basal forebrain cholinergic neurons, which innervate the hippocampus, cortex, and other brain regions, play a critical role in cognition, arousal, and integration of brain responses (Mesulam et al., 1983; Baxter and Chiba, 1999). Although we did not determine cognition or other behaviors in these experiments, AIE has been found to cause lasting changes in adult behavior, including deficits in behavioral flexibility (Vetreno and Crews, 2015; Crews et al., 2019; Vetreno et al., 2019). Studies find that prevention of AIE-induce neuroimmune signaling with indomethacin or wheel running exercise prevents AIE-induced reversal learning deficits, an index of behavioral flexibility as well as loss of BFCN markers (Pascual et al., 2007; Vetreno and Crews, 2018). Recently, we reported that exercise restored the AIE-induced reversal learning deficits in association with reversal of increased pNF-κB p65 expression and histone gene silencing markers (Vetreno et al., 2019) similar to those reported here. The exercise restoration of AIE-induced behavioral flexibility deficits and loss of BFCN markers, coupled with reversal of neuroimmune signaling links AIE neuroimmune induction and cognitive deficits to epigenetic silencing and loss of cholinergic neurons. Consistent with cholinergic dysfunction contributing to behavioral deficits, Wang et al. (2019) reported that choline supplementation blunted the loss of ChAT + BFCNs and subsequent cognitive deficits in the APP/PS1 mouse model of AD. Other studies focused on AIE-induced increases in adult anxiety and alcohol drinking find AIE epigenetic programming in amygdala that can be reversed, with associated reversal of increased anxiety-like behavior and alcohol drinking (Pandey et al., 2015; Sakharkar et al., 2016). Swartzwelder et al. (2019) reported AIE-induced reductions of hippocampal neurogenesis were accompanied by increased expression of RAGE and pNF-kBp65 as well as H3K9me2 that was reversed by treatment with the cholinesterase inhibitor donepezil in adulthood. Donepezil has also been shown to reverse epigenetic modification of *Fmr1* in the hippocampus of adult AIE-treated rats (Mulholland et al., 2018). Recent studies find transgenic mice lacking TLR4 do not show AIE-induced mPFC changes in histone acetylation and methylation (Montesinos et al., 2016), consistent with neuroimmune signaling altering gene expression, in part through epigenetic mechanisms. In the present study, galantamine treatment post-AIE reversed the AIE increase of HMGB1-RAGE/TLR4-pNF-κB p65, recovered the increased HeK9me2 associated with *ChAT* and *TrkA* promoters, and restored the loss of BFCNs in the adult basal forebrain that likely restores AIE behavioral pathology, although not directly assessed in this study.

In conclusion, treatment with the cholinesterase inhibitor galantamine, either during AIE or at the conclusion of AIE, recovered the loss of BFCNs and concomitant HMGB1-RAGE/TLR4-pNF-κB p65 neuroimmune activation in the adult basal forebrain. We report BFCNs express TLR4, RAGE, and activated pNF-κB p65, and AIE treatment increased pNF-κB p65 expression in adult ChAT + IR neurons that was recovered by galantamine treatment, consistent with neuroimmune activation in BFCNs. The lack of NeuN + IR neuronal cell loss and the restorative effects of post-AIE galantamine treatment on BFCNs suggest the loss of the cholinergic neuron phenotype and not a cell death mechanism. The AIE-induced loss of cholinergic neuron markers was accompanied by an increase of cellular histone 3 methylation markers (i.e., H3K9me2 and H3K9me2) as well as a persistent increase of histone methylation at promoter regions of both the *ChAT* and *TrkA* gene, which were recovered by galantamine treatment in AIE-treated subjects. Together, these data suggest that AIE induces a novel neuroplastic process involving neuroimmune signaling and epigenetic gene silencing within BFCNs that results in the loss of the cholinergic neuron phenotype that can be recovered by galantamine treatment.

## Data Availability Statement

The raw data supporting the conclusions of this article will be made available by the authors, without undue reservation.

## Ethics Statement

The animal study was reviewed and approved by the Institutional Animal Care and Use Committee of the University of North Carolina at Chapel Hill.

## Author Contributions

FC and RV were responsible for the study concept and design. All authors contributed equally to the data preparation and analysis, involved in drafting and editing the manuscript, and approved the final manuscript version for publication.

## Conflict of Interest

The authors declare that the research was conducted in the absence of any commercial or financial relationships that could be construed as a potential conflict of interest.
